# Development of a touchdown droplet digital PCR assay for the detection and quantitation of human papillomavirus 16 and 18 from self-collected anal samples

**DOI:** 10.1128/spectrum.01836-23

**Published:** 2023-11-14

**Authors:** Gholamreza Haqshenas, Suzanne M. Garland, Prisha Balgovind, Alyssa Cornall, Jennifer Danielewski, Monica Molano, Dorothy A. Machalek, Gerald Murray

**Affiliations:** 1 Department of Obstetrics and Gynaecology, University of Melbourne, Melbourne, Victoria, Australia; 2 Centre for Women’s Infectious Diseases, The Royal Women’s Hospital, Melbourne, Victoria, Australia; 3 Murdoch Children’s Research Institute, Melbourne, Victoria, Australia; 4 The Kirby Institute, UNSW Sydney, Sydney, New South Wales, Australia; Quest Diagnostics, Chantilly, Virginia, USA

**Keywords:** human papillomavirus, droplet digital PCR, anal samples

## Abstract

Broad-spectrum droplet digital polymerase chain reaction (ddPCR) assays can be used to quantitate multiple human papillomavirus (HPV) types. However, the presence of nucleotide mismatches at the primer binding sites may result in different optimal annealing temperatures and, consequently, under detection of some HPV types. Touchdown PCR uses decreasing annealing temperature over the first PCR cycles allowing for specific and efficient amplification of the target sequence. Here, as a proof-of-concept, we assessed if touchdown ddPCR (TD-ddPCR) using broad-spectrum oligonucleotides and genotype-specific probes targeting HPV L1 can be used to quantitate HPV. We tested our hypothesis using HPV positive cell lines and anal samples which potentially contain PCR inhibitors. Using serial dilutions of HPV16 and HPV18 DNA, the TD-ddPCR assays were performed with linearity across the dilution series (Pearson’s correlation r = 0.9999) and detected one copy of both HPV types, with no cross-reactivity to 27 other low-risk and high-risk HPV types. The PCRs were highly reproducible, with inter- and intra-assay coefficient of variations ranging 2.0–6.3% for HPV16 and 4.9–11.6% for HPV18. Testing self-collected anal samples positive for HPV16 (*n* = 29) or HPV18 (*n* = 33) and those negative for both HPV types (*n* = 22) with the ddPCR assay and Seegene Anyplex II HPV28 (Seegene, TD-ddPCR) showed high agreements between the two assays. In conclusion, HPV broad-spectrum primers containing mismatches at their target binding sites and a touchdown thermal cycling approach resulted in sensitive and specific amplification of HPV genome using ddPCR. The assay has potential clinical diagnostic applications for detection, quantitation, and monitoring of anal HPV infections.

**IMPORTANCE:**

The quantity of the human papillomavirus (HPV) is associated with disease outcome. We designed an accurate and precise digital PCR assay for quantitating HPV in anal samples, a sample type that is typically problematic due to the presence of PCR inhibitors.

## INTRODUCTION

Human papillomavirus (HPV) infection may result in a variety of benign and aggressive neoplastic lesions on the skin or mucosa of anogenital tract and oropharynx (1 – 4). HPV, a member of the Papillomaviridae family, contains a double-stranded circular DNA genome (~8 kb) which encodes two late structural (L1 and L2) proteins that form the viral capsid and six early nonstructural (E1, E2, E4, E5, E6, and E7) proteins that promote viral replication and host cell transformation (4). The International Agency for Research on Cancer (IARC) has classified 12 HPV types (HPV16, HPV18, HPV31, HPV33, HPV35, HPV39, HPV45, HPV51, HPV52, HPV56, HPV58, and HPV59) as Group 1 carcinogens and one (HPV68) as potentially carcinogenic out of the more than 200 HPV types that have been reported (5). HPV16 is the most oncogenic and, together with HPV18, have been found in the vast majority of invasive HPV-associated cancers globally in humans, including anogenital cancers (6 – 10) and oropharyngeal cancers (11).

HPV16 and HPV18 are associated with 84.3% of anal cancers (6). Anal cancer incidence rate is increasing annually in some countries such as the US (12). According to the 2020 Global Cancer Statistics report, anal cancer accounts for 0.3% (or 50,865 people) of all cancer cases worldwide (13). Anal cancer is less common than cervical cancer but causes mortality in both men and women (13). It occurs more frequently in at-risk groups [e.g., men who have sex with men (MSM) and compromised individuals]. A recent IARC meta-analysis assessment on 29,900 MSM shows that up to 28.5% were HIV and HPV positive, and 10% of these had high-grade squamous intraepithelial lesions (HSIL) (14), a potential precursor to invasive anal lesions.

Consistent with other HPV-associated cancers (15, 16), several studies have shown a direct correlation between viral load and anal cancer survival rate (17 – 19). Also, HPV viral load has been reported as a predictive biomarker for anal HSIL (20). The above reports highlight the need for the development of accurate quantitative assays for high-risk (HR) HPV types 16 and 18.

Although there are many commercially available HPV detection kits, the majority are not quantitative assays (21). The currently available quantitative PCR (qPCR) methods are highly susceptible to the presence of contaminants, in particular, the high level of PCR inhibitors found in anal samples (22). Also, the qPCR-based tests rely on a standard curve which is used to generate a relative quantitation value based on the copy numbers used for each data point in the standard curve. Droplet digital PCR (ddPCR) is the third generation of PCR which enables absolute quantitation of specific target sequences and is less sensitive to contaminants that can act as PCR inhibitors (22 – 24). Using conventional PCR thermal cycles and genotype-specific primers and probes, several research groups have used ddPCR for the detection of HPV in anogenital or oropharyngeal samples (17, 22, 25 – 33). Broad-spectrum primers are suitable for the development of multiplex tests because they allow for the simultaneous amplification of several HPV types. These primers do, however, have mismatches at their binding sites, which necessitate lower annealing temperatures for effective amplification potentially leading to off-target amplification. Here, we developed a touchdown ddPCR (TD-ddPCR) assay for the detection and quantitation of HPV16 and HPV18 from anal samples utilizing broad-spectrum primers and genotype-specific probes in conjunction with touchdown thermal cycles (decreasing annealing temperature over the first PCR cycles).

## MATERIALS AND METHODS

### HPV control samples

SiHa (ATCC HTB35) (34) and HeLa (ATCC CCL2) (35) cell lines containing HPV16 (1–2 viral genome copies per cell) or HPV18 (10–50 viral genome copies per cell), respectively, were used for optimization of the assay (36). World Health Organization (WHO) HPV DNA standards of genotypes 16 (NIBSC code 06/202) and 18 (NIBSC code 06/206) (37) were purchased from WHO. The standards contain HPV full genomes in plasmid backbones at 10^7^ HPV genomic equivalents per milliliter.

### Clinical samples

Eighty-four self-collected anal swab samples were from the National HPV Monitoring Program (IMPACT), an ongoing surveillance program for monitoring the impact of nationwide vaccination on the prevalence of HPV types in Australia’s population (38, 39). Samples were collected in Cobas PCR medium (Roche) or Amies transport medium. The study was conducted with ethical approval from Royal Melbourne Hospital Human Research Ethics Committee (HREC/59265/MH-2019).

### Nucleic acid (NA) extraction

HeLa and SiHa cells (10^7^ cells each) were pelleted separately by centrifugation at 1,500 × *g* for 5 minutes at room temperature. Cell pellets were resuspended in 200 µL of phosphate-buffered saline (pH 7.4). Using the Roche MagNA Pure 96 DNA and Viral NA Small (or Large) Volume Kit (MP96, Roche), we extracted total NA from cell lines (200 µL) and anal samples (500 µL) (40) then eluted them in 100 µL and 50 µL of Roche elution buffer, respectively. Using qPCR, we measured the human β-globin gene as an indication of the integrity of extracted DNA and to estimate cell number (each cell contains two copies of the β-globin gene) (10, 41). The purified NA from SiHa and HeLa cells were quantified using a NanoDrop 2000. All extracted NA samples were stored at −30°C.

### HPV genotyping

Anal swab samples were tested using Seegene Anyplex II HPV28 (Anyplex28; Seegene, Seoul, South Korea) as described previously (10, 42 – 44). The Anyplex28 assay detects 28 HPV genotypes being deemed as HR (16, 18, 31, 33, 35, 39, 45, 51, 52, 56, 58, 59, 66, and 68) or potential HR and low-risk (6, 11, 26, 40, 42, 43, 44, 53, 54, 61, 69, 70, 73, and 82). The assay is semiquantitative and reports each HPV genotype with a crossing point (Cq) range, ≤31 cycles (+++), 31–39 cycles (++), and ≥40 cycles (+). This Cq range is also used by the assay to assess the validity of the results; samples with an internal control (IC) Cq ≥ 40 or undetected are defined as invalid.

### Droplet digital PCR

Previously reported modified versions of GP5+/6+ (MGP) primers with a 10-base stretch at their 5' ends were used (45) (Table S1). These broad-spectrum primers amplify a conserved DNA segment (~150 bp) within the HPV L1 gene (Fig. S1). Two specific probes were designed for HPV types 16 and 18 within the amplicon sequences (Table S1; Fig. S2). The probes were designed to be highly conserved across HPV16 and HPV18 sequences available in GenBank and displayed absolute identity with 99% of the HPV16 or HPV18 sequences (617/622 and 145/146, respectively). The remaining sequences contain only one mismatch at their binding sites. These were labeled with either FAM or HEX fluorophores containing an internal ZEN Quencher and 3' Iowa Black Fluorescent Quencher (Integrated DNA Technologies). The positions of primers and probes used in this study were mapped on HPV reference sequences obtained from the PaVE (Papilloma Virus Episteme) (Fig. S1 and S2).

Each ddPCR reaction contained 10 µL of 2× ddPCR Supermix for Probes (No dUTP) (BioRad), 500 nM each of the three primers (MGPB, MGPC, and MGPH), 250 nM probe, and 2–5 μL of DNA extracts in a final volume of 20 µL. Droplets were generated with a BioRad QX200 Droplet Generator following the manufacturer’s instructions. The standard two-step ddPCR and touchdown (decreasing 2°C per two cycles over five cycles) thermal cycles were used for assay optimization (Table S2). Reactions were analyzed using the QX100 Droplet Reader (BioRad). The HPV genome copy number was quantified using the QuantaSoft Software which included a Poisson distribution correction step (46). Positive droplets were determined using a threshold value relative to the negative control.

To assess the reproducibility of the TD-ddPCR assays, we estimated intra-assay coefficient of variation using triplicates of SiHa or HeLa cell extracts and both FAM- and HEX-labeled probes. Standard deviations and averages were calculated and used to estimate intra-assay coefficient of variations for triplicate samples of SiHa and HeLa cells. Likewise, to calculate the inter-assay coefficient of variation, we evaluated the average of the above three measures and the results of two additional TD-ddPCR reactions (performed on two different occasions) for both HPV16 and HPV18 (FAM or HEX).

The sensitivity of each assay, minimum detectable HPV copies by the assays, was evaluated using quantitated WHO DNA standards of HPV16 and HPV18. Four 10-fold serial dilutions (1/10, 1/100, 1/10,000, and 1/100,000) of the DNA standards were prepared in Tris-EDTA buffered saline (10 mM Tris-HCl and 1 mM EDTA, pH 8) and tested in duplicate using TD-ddPCR and both the FAM- and HEX-labeled probes. To measure the HPV genomic copy number per SiHa and HeLa cells, we used a combination of specific primers and FAM-labeled probes for human β-globin (47) and HPV primers and HEX-labeled probes (Table S1) in a single reaction.

### Statistical analysis

For assay comparison, the overall agreements, negative and positive percent agreements (NPA and PPA, respectively), and Cohen’s kappa statistic were calculated (40, 48). Intra- and inter-assay coefficient of variations (CV) (49) and assay precision for both assays were calculated. For comparison of the results obtained from using genotype-specific probes labeled with different fluorophores (FAM and HEX), two-sided paired Student’s *t*-test (Microsoft Excel) was used and *P* < 0.05 was deemed significant. GraphPad Prism version 9.1.1 for Windows (GraphPad Software, San Diego, California USA) was used to analyze the ddPCR HPV quantitation results from Anyplex28 HPV positive clinical samples. Microsoft Excel was used for the calculation of Pearson’s correlation.

## RESULTS

### Optimization of droplet digital PCR conditions

**Fig 1:**
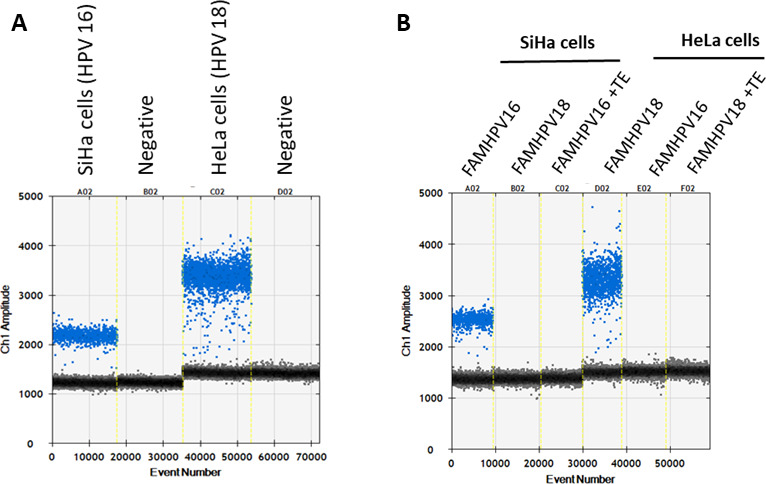
Quantitation of HPV16 and HPV18 using touchdown droplet digital PCR. Three broad-spectrum HPV primers and genotype-specific FAM-labeled probes (see Materials and Methods) were used to detect and quantify HPV16 and HPV18 from SiHa and HeLa cells, respectively. Negative and positive droplets are depicted as gray and colored, respectively. No probe cross-reactivity was observed (**B**).

As mentioned above, the broad-spectrum primers used in this study contained several nucleotide differences with their target HPV sequences that could potentially affect their optimal annealing temperatures. Using SiHa and HeLa cell lines harboring HPV type 16 (1–2 copies per cell) or 18 (10–50 copies per cell) genomes, our initial attempts using the standard two-step thermal cycles (Table S2) failed to generate any positive droplets (data not shown). We then tested a three-stepwise (decreasing/increasing temperatures) cycling program toward a defined annealing temperature (Table S2). The annealing temperatures were based on that previously described (45) whereby 42°C was used for the first five cycles, followed by 45 cycles at 64°C. The touchdown conditions using decremental annealing temperatures from 50°C to 42°C (decreasing 2°C per cycle over five cycles) resulted in positive droplets using the HPV control SiHa or HeLa cells (Fig. 1A). Using incremental thermal cycles from 42°C to 48°C (increasing 2°C per cycle over four cycles) resulted in the same results (data not shown). Simultaneous detection of HPV and β-globin showed no remarkable effect on their detection levels and detected approximately three and eight copies of HPV types 16 and 18 genomes per cell of SiHa and HeLa lines, respectively.

### Reproducibility of TD-ddPCR

To show the reproducibility of the results, using SiHa and HeLa cells, we calculated intra- and inter-assay coefficients of variation for HPV16 and HPV18 using FAM- and HEX-labeled probes for each target (Table 1). There was not a significant difference between using FAM- and HEX-labeled probes for quantitation of HPV16 (*P* = 0.46) or HPV18 (*P* = 0.06).

**TABLE 1:** Reproducibility of digital PCR assays^*a*^

	HPV16		HPV18	
	FAM	HEX	FAM	HEX
Intra-assay CV (%)	9.5	10.0	4.9	11.6
Inter-assay CV (%)	5.1	3.3	2.0	6.3

^*a*^ Triplicate results were used to calculate intra- and inter-assay coefficients of variation.

### Precision and cross-reactivity of TD-ddPCR assays

Precision was assessed using 10-fold serial dilutions of WHO HPV16 and HPV18 international NA standards. TD-ddPCR detected down to one genome copy of HPV16 and HPV18 (Table 2), although at higher concentrations than one copy, it reported approximately half of the calculated copy number of the standards. The test was performed with linearity across the dilution series. Performance was the same using either FAM- or HEX-labeled probes. A strong Pearson’s correlation (r = 0.9999) was observed between the input concentrations of standard DNA samples and the obtained ddPCR measurements for HPV16 and HPV18 using FAM or HEX probes.

**TABLE 2:** Precision of the ddPCR assays^*a*^

WHO standard	HPV16 FAM	HPV16 HEX	HPV18 FAM	HPV18 HEX
1000 IU	440.5 ± 40.3	455.0 ± 14.1	353.0 ± 15.6	343.5 ± 12.0
100 IU	41.0 ± 4.9	44.5 ± 4.9	39.5 ± 4.9	37.5 ± 4.9
10 IU	4.0 ± 0.1	6.3 ± 0.1	3.3 ± 0.1	2.5 ± 1.6
1 IU	1.5 ± 1.0	0.7 ± 1.0	1.1 ± 0.6	0.0 ± 0.0

^*a*^ Serial dilutions of WHO standards were prepared in Tris-EDTA buffer (TE) and tested by ddPCR using FAM- and HEX-labeled HPV16 or HPV18 probes. The averages of duplicates are presented with ±standard deviations.

To assess the cross-reactivity of each assay, initially we used DNA extracts from SiHa and HeLa cell lines containing HPV16 or HPV18 genomes. As shown in Fig. 1B, no cross-reactivity was observed when the HPV16 probe was used against HPV18 template and vice versa. We then used anal swab samples tested by Anyplex28 strongly positive (3+) for 26 HPV genotypes including the 12 known HR HPVs (HPV16, HPV18, HPV31, HPV33, HPV35, HPV39, HPV45, HPV51, HPV52, HPV56, HPV58, HPV59). Using HPV positive anal samples, none of the tested 27 genotypes cross-reacted with assays for detection of HPV16 or HPV18. Notably, FAM-labeled probes produced higher background noise in HPV negative samples (Fig. S3); thus, further experiments were conducted using HEX-labeled probes.

### Performance of ddPCR for the detection of HPV from anal samples

**Fig 2:**
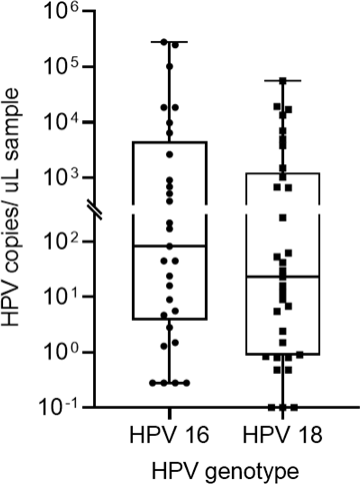
Quantitation of HPV16 (*n* = 29) and HPV18 (*n* = 33) from Anyplex28 positive anal samples using TD-ddPCR. The horizontal lines of the box-and-whisker plots represent the median, and the whiskers represent the minimum and maximum values.

Overall, 84 samples tested by Anyplex28 as HPV16/18 negative (*n* = 22), HPV16 positive (*n* = 29), and HPV18 positive (*n* = 33) with valid IC signals (++ and +++) were analyzed by ddPCR (Table 2). The results of testing the samples for HPV16 on Anyplex28 HPV16 and TD-ddPCR (Tables 3 and 4) showed NPA and PPA of 100% [95% confidence interval (CI), 84.6–100.0] and 100% (95% CI, 88.1–100.0), respectively. The level of HPV detection ranged from 0.28 to 2.8 × 10^5^ genomes per microliter of sample (median, 83) (Fig. 2). Of the 22 Anyplex28 negative samples, one was positive for HPV18 using HEX probe (Fig. S3), containing 20 HPV genomes per microliter of extract (Table 3). For HPV18 detection, 30/33 (90%) of samples were positive on the TD-ddPCR (Table 4), with an NPA of 95.5% (95% CI, 77.2–99.9) and a PPA of 90.9% (95% CI, 75.7–98.1) compared to Anyplex28. The level of HPV detection ranged from 0 to 5.5 × 10^4^ copies per microliter of sample (median, 23) (Fig. 2). Two of the three samples that were negative by TD-ddPCR and had low Anyplex28 signal (+) were confirmed by retesting on the Anyplex28. Calculation of kappa values between the two assays for detection of HPV16 and HPV18 showed a substantial agreement for both HPV16 (0.755; 95% CI, 0.591–0.920) and HPV18 (0.690; 95% CI, 0.509–0.871). The overall agreements between the two assays were 100% (95% CI, 93.0–100.0) and 92.7% (95% CI, 82.4–98.0) for HPV16 and HPV18, respectively (Table 4).

**TABLE 3:** Detection of HPV16 and HPV18 from anal samples using Anyplex28 and TD-PCR

Testing anal Anyplex28 HPV positive samples by TD-ddPCR					
HPV type tested	Total samples	No. of samples	Anyplex28 HPV score	TD-ddPCR results	
				Positives	HPV copies^*a*^ (median)
HPV16	29	7	+	7	0.28–4.7 (0.9)
		14	++	14	1.3–18,570 (45)
		8	+++	8	379–278,500 (9,770)
HPV18	33	6	+	4	0–23 (0.64)
		15	++	15	0.48–62 (7.9)
		12	+++	11	0–55,200 (3,800)
HPV16	22^*b*^	0	0/22	0	NA
HPV18	22^*b*^	0	0/22	1	20

^*a*^ Copies per microliter of input sample.

^*b*^ HPV16/18 negative samples.

**TABLE 4:** Overall assay agreement between Anyplex28 and touchdown (TD-ddPCR) for the detection of HPV16 and 18^*a*^

			Seegene Anyplex28		
			Positive	Negative	Total
TD-ddPCR	HPV16	Positive	29	0	29
		Negative	0	22	22
		Total	29	22	51
	HPV18	Positive	30	1	31
		Negative	3	21	24
		Total	33	22	55

^*a*^ These values were used to calculate negative and positive percent agreements.

## DISCUSSION

In this study, we developed a TD-ddPCR protocol using broad-spectrum HPV primers and genotype-specific probes for HPV types 16 and 18, which detected as few as one copy of HPV genome and did not cross-react with any of the 26 non-HPV16/18 types, indicating high precision and accuracy. Anyplex28 and TD-ddPCR produced 100% concordant results when 51 anal swab samples were tested for HPV16. A slightly lower agreement was observed for HPV18 (95% PPA and 91% NPA).

Development of broad-spectrum PCR assays combined with genotype-specific probes can facilitate the development of multiplex HPV detection assays. However, genetic diversity across HPV types with less than 90% similarities (50) is a major hurdle to achieving this. Among HPV genes, the L1 gene is relatively conserved across HR HPV types and, therefore, this region has been used for designing broad-spectrum PCRs (45, 51 – 53). The first broad-spectrum HPV PCR utilized two relatively conserved primers (GP5+/GP6+) within the L1 gene of HR HPV types (51). Improvements include touchdown cycling and inclusion of degenerate bases to increase the breadth of HPV type coverage (54). The addition of a 10-base stretch at the 5' end (MGP primers) allowed high annealing temperatures after initial PCR cycles, enhancing detection (45). The MGP primers detect all 14 HR HPV types with high sensitivity (five copies/PCR), but their use in digital PCR has not been explored. This study developed TD-ddPCR assays using the selected MGP primers and genotype-specific probes for the detection and quantitation of HPV16 and HPV18 from anal samples, which are generally challenging samples for PCR-based detection techniques due to the presence of potential PCR inhibitors in feces (55). Digital PCR is generally a two-step PCR with the same annealing and extension temperatures, i.e., 60°C. Here, we used three-step TD-ddPCR and demonstrated that this approach could be applied successfully to ddPCR technology for HPV detection in clinical samples and cell lines. The use of a three-step TD-PCR has been reported for the detection and quantitation of HIV sequences (56).

The TD-ddPCR assays described in this study detected three and eight HPV genomes per each SiHa and Hela cell, respectively, which slightly differed from the known figures (1–2 and 10–50 HPV copies per SiHa and HeLa cell, respectively), and this discrepancy could be due to inter-laboratory variation in sample preparation and the cells used in each laboratory. When tested against the quantitated WHO standards, the TD-ddPCR assays detected fewer HPV genome copies than expected; such discrepancies have been reported previously (12, 57 – 59). Using an independent ddPCR targeting HPV E6 resulted in similar values (data not shown). This could be due to the degradation of the DNA standards and inter-laboratory technical differences.

The HPV18 TD-ddPCR did not detect HPV in three samples that were HPV18 positive by Seegene Anyplex28. Of these, two had 1+ Anyplex28 signal (Ct < 40) and one had 3+ signal. PCR amplification and Sanger sequencing of the entire L1 and L2 genes of the HPV18 sample with an Anyplex28 3+ signal showed a large deletion containing the MGP binding site (independent manuscript in preparation), but the other two samples were inadequate for PCR amplification and sequencing. The failure to detect the remaining two samples by ddPCR could be due to the presence of nucleotide mismatches at primer binding sites or lower viral titer; lower sensitivity of ddPCR relative to other detection assays has been reported previously for clinical samples (27).

Study limitations include using only one type of sample, although pilot experiments showed that the TD-ddPCR can be used for the detection of HPV in cervical and formalin-fixed paraffin-embedded specimens (data not shown). The assays were developed for the detection of just two HPV types (16 and 18), one at the time, from limited numbers of clinical samples, although these are by far the most important HPV types. The described methodology can be applied for the detection of other HPV types, but further studies are required to select combinations of appropriate fluorophores for the development of multiplex assays for simultaneous detection and quantitation of HPV types.

In conclusion, the TD-ddPCR assay is a highly sensitive method with no cross-reactivity with other genotypes for the detection and absolute quantification of HPV in anal swab samples. These assays provide a method for assessing and monitoring HPV viral load over the course of infection and treatment. Additionally, due to the use of broad-spectrum primers, this test forms a platform for a multiplex TD-ddPCR for the detection of several HPV types from one small sample in one reaction. Additionally, the touchdown approach described here can be applied to any ddPCR aimed at the detection of a heterogenetic population of genomes with slight sequence variations at primer binding sites.

## SUPPLEMENTAL MATERIAL

Supplemental file 1 spectrum.01836-23-s0001.tif Fig. S1.

Supplemental file 2 spectrum.01836-23-s0002.tif Fig. S2.

Supplemental file 3 spectrum.01836-23-s0003.tif Fig. S3.

Supplemental file 4 spectrum.01836-23-s0004.docx Table S1.

Supplemental file 5 spectrum.01836-23-s0005.docx Table S2.

Supplemental file 6 spectrum.01836-23-s0006.docx Legends to Fig. S1 to S3.

ASM does not own the copyrights to Supplemental Material that may be linked to, or accessed through, an article. The authors have granted ASM a non-exclusive, world-wide license to publish the Supplemental Material files. Please contact the corresponding author directly for reuse.
