# Supplementary figures and images for: Development of a touchdown droplet digital PCR assay for the detection and quantitation of human papillomavirus 16 and 18 from self-collected anal samples
Source: Microbiol Spectr. 2023 Nov 14;11(6):e01836-23. doi: 10.1128/spectrum.01836-23 (PMC10714734; doi:10.1128/spectrum.01836-23)

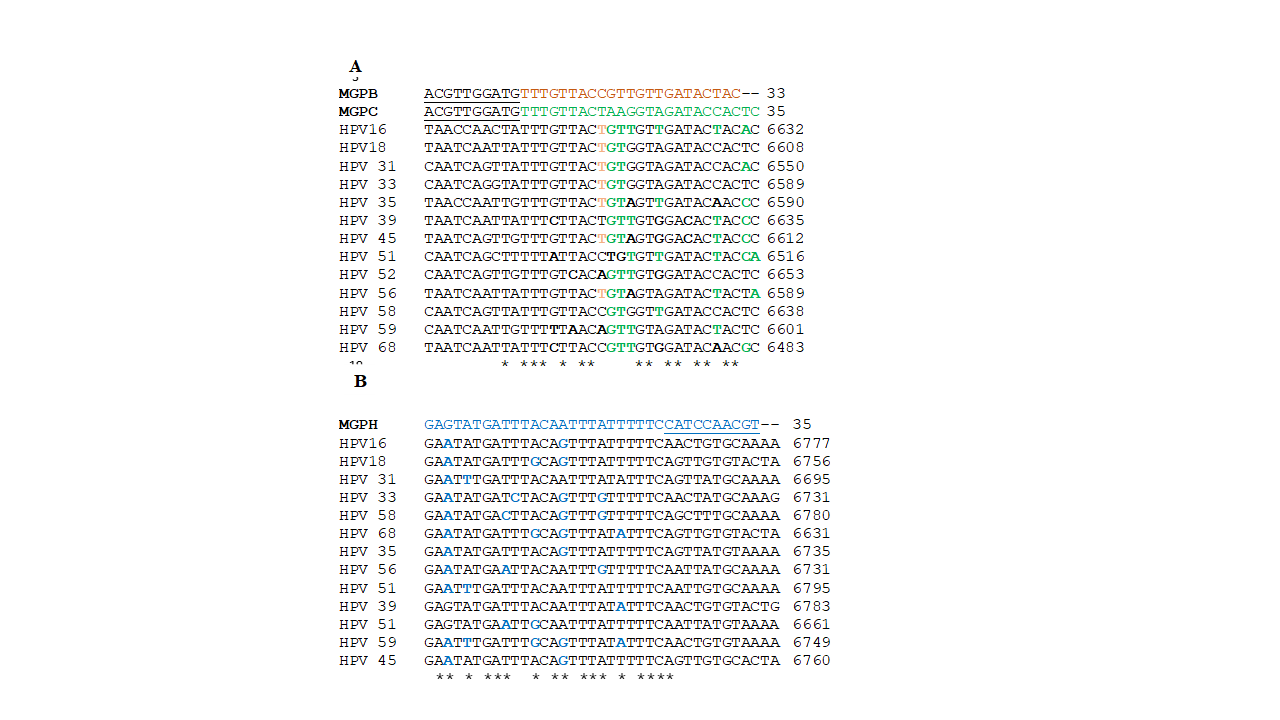

Supplement: Supplemental file 1 — Fig. S1. [file spectrum.01836-23-s0001.tif]

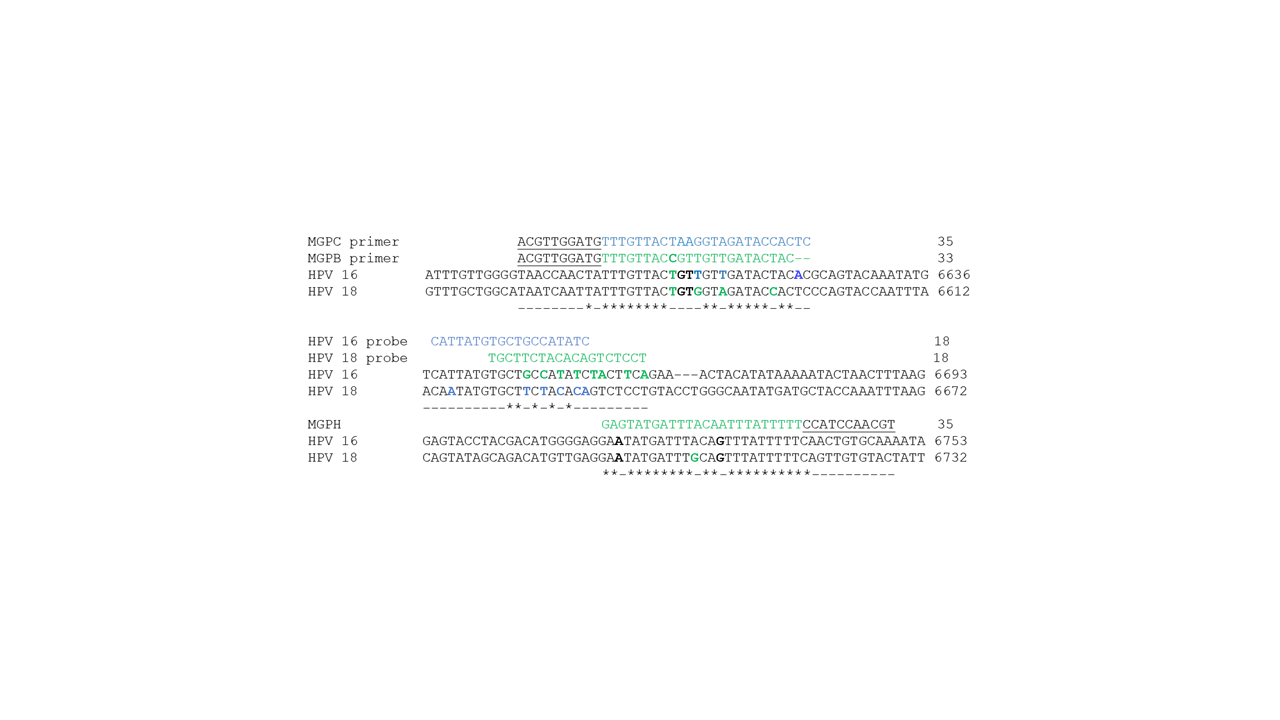

Supplement: Supplemental file 2 — Fig. S2. [file spectrum.01836-23-s0002.tif]

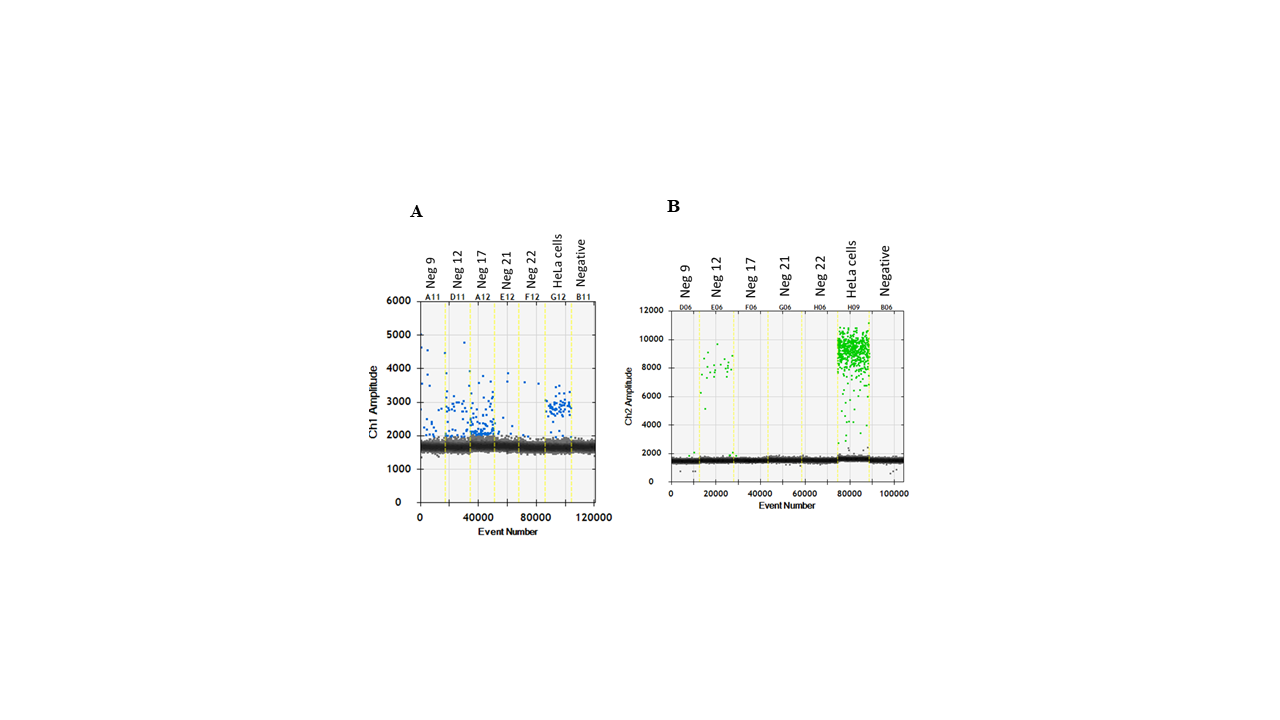

Supplement: Supplemental file 3 — Fig. S3. [file spectrum.01836-23-s0003.tif]
