# Supplementary material for: Development of a touchdown droplet digital PCR assay for the detection and quantitation of human papillomavirus 16 and 18 from self-collected anal samples
Source: Microbiol Spectr. 2023 Nov 14;11(6):e01836-23. doi: 10.1128/spectrum.01836-23 (PMC10714734; doi:10.1128/spectrum.01836-23)
Supplement: Supplemental file 4 — Table S1. [file spectrum.01836-23-s0004.docx]

**Supplemental Table 1**. List of oligonucleotide primers and probes used in this study.

| Name | Target gene | Function | Direction | Nucleotide sequence^a^ |
| --- | --- | --- | --- | --- |
| MGPB | HPV L1 | primer | Sense | **ACGTTGGATG**TTTGTTACCGTTGTTGATACTAC |
| MGPC | HPV L1 | primer | Sense | **ACGTTGGATG**TTTGTTACTAAGGTAGATACCACTC |
| MGPH | HPV L1 | primer | Antisense | **ACGTTGGATG**GAAAAATAAATTGTAAATCATACTC |
| FAMHPV16^b^ | HPV L1 | probe | Sense | FAM CATTATGTGCTGCCATATC/3BHK |
| HEXHPV16^b^ | HPV | probe | Sense | HEX CATTATGTGCTGCCATATC/3BHK |
| FAMHPV18^b^ | HPV | probe | Sense | FAM TGCTTCTACACAGTCTCCT/3BHK |
| HEXHPV18^b^ | HPV L1 | probe | Sense | HEX TGCTTCTACACAGTCTCCT/3BHK |
| PCO3^c^ | β-globin | Primer | Sense | ACACAACTGTGTTCACTAGC |
| PCO4^c^ | β-globin | Primer | Antisense | CCACTTGCACCTACTTCAAC |
| RS06^c^ | β-globin | Probe | Sense | CTGACTCCTGAGGAGAAGTCTGCCGTTACTGCCCTGTGGG |

^a^ Bolded nucleotides are non-HPV sequences. ^b^ reported by Soderlund-Strand et al., 2009. ^c^ reported by Saiki et al., 1985.
