# Supplementary material for: Development of a touchdown droplet digital PCR assay for the detection and quantitation of human papillomavirus 16 and 18 from self-collected anal samples
Source: Microbiol Spectr. 2023 Nov 14;11(6):e01836-23. doi: 10.1128/spectrum.01836-23 (PMC10714734; doi:10.1128/spectrum.01836-23)
Supplement: Supplemental file 5 — Table S2. [file spectrum.01836-23-s0005.docx]

**Supplemental Table 2**. Thermal cycles used in this study.

| Thermal  cycles | Polymerase activation (10 min) | Initial annealing (10 sec) | Annealing/  Extension (1 min) | Number of cycles | Final  (10 min) |
| --- | --- | --- | --- | --- | --- |
| Fixed | 95 ºC | - | 60 ºC^a^ | 50 | 98 ºC |
| Touchdown | 95 ºC | 50-42ºC (reducing 2ºC every two cycles) | 60 ºC^c^ | 45 | 98 ºC |
