# Supplementary material for: Development of a touchdown droplet digital PCR assay for the detection and quantitation of human papillomavirus 16 and 18 from self-collected anal samples
Source: Microbiol Spectr. 2023 Nov 14;11(6):e01836-23. doi: 10.1128/spectrum.01836-23 (PMC10714734; doi:10.1128/spectrum.01836-23)
Supplement: Supplemental file 6 — Legends to Fig. S1 to S3. [file spectrum.01836-23-s0006.docx]

**Supplementary Figure 1**. Alignment of 13 high-risk HPV reference sequences with MGP forward (A, MGPB and MGPC) and reverse (B, MGPH) primers. A) Brown and green nucleotides differ from MGPB and MGPC, respectively, and black-bolded nucleotides differ from both primers. Right column shows nucleotide positions in HPV full length genome sequences for primers. HPV16 (K02718), HPV18 (X05015), HPV 31 (J04353), HPV 33 (M12732), HPV 35 (X74477), HPV 39 (M62849), HPV 45 (X74479), HPV 51 (M62877), HPV 52 (X74481), HPV 56 (X74483), HPV 58 (D90400), HPV 59 (X77858), HPV 68 (DQ080079).

**Supplementary Figure 2.** Alignment of HPV16 and HPV18 primers and probes with reference genomes. The nucleotide positions are depicted on the right-hand side. Bolded black nucleotides differ from both primers, while bolded color nucleotides differ from corresponding aligned primer.

**Supplementary Figure 3**. Testing Anyplex28 HPV18 negative samples on TD-ddPCR assays using the FAM- and HEX-labelled probes. Some samples (e.g., Neg 9, Neg 12, Neg 17, Neg 21, and Neg 22) and negative produced different levels of positivity using FAM-labelled probes samples (A). Testing the above samples using the HEX-labelled probe, only N12 was positive using TD-ddPCR. HeLa cells extract was used as positive control.
